# The evolutionary species pool concept does not explain occurrence patterns of dead-wood-dependent organisms: implications for logging residue extraction

**DOI:** 10.1007/s00442-019-04473-2

**Published:** 2019-07-27

**Authors:** Thomas Ranius, Aino Hämäläinen, Jörgen Sjögren, Matthew Hiron, Dennis Jonason, Ariana Kubart, Martin Schroeder, Anders Dahlberg, Göran Thor, Mats Jonsell

**Affiliations:** 1 Department of Ecology, Swedish University of Agricultural Sciences, Box 7044, SE-750 07 Uppsala, Sweden; 2 Department of Wildlife, Fish and Environmental Studies, Swedish University of Agricultural Sciences, 901 83 Umeå, Sweden; 3 Department of Forest Mycology and Pathology, Swedish University of Agricultural Sciences, Box 7026, SE-750 07 Uppsala, Sweden

**Keywords:** Beetles, Fungi, Lichens, Slash, Woody debris

## Abstract

Emulation of natural disturbances is often regarded as a key measure to make forestry biodiversity-oriented. Consequently, extraction of logging residues is assumed to have little negative effect in comparison to extraction of dead wood mainly formed at natural disturbances. This is consistent with the evolutionary species pool hypothesis, which suggests that most species are evolutionary adapted to the naturally most abundant habitats. We tested this hypothesis for dead-wood-dependent macrofungi, lichens, and beetles in a boreal forest landscape in central Sweden, assuming that species are adapted to conditions similar to today’s unmanaged forest. No occurrence patterns, for the species groups which we investigated, were consistent with the hypothesis. Overall, stumps and snags had the highest habitat quality (measured as average population density with equal weight given to each species) and fine woody debris the lowest, which was unexpected, since stumps were the rarest dead-wood type in unmanaged forest. We conclude that the evolutionary species pool concept did not explain patterns of species’ occurrences, and for two reasons, the concept is not reliable as a general rule of thumb: (1) what constitute habitats harbouring different species communities can only be understood from habitat-specific studies and (2) the suitability of habitats is affected by their biophysical characteristics. Thus, emulation of natural disturbances may promote biodiversity, but empirical studies are needed for each habitat to understand how natural disturbances should be emulated. We also conclude that stump extraction for bioenergy is associated with larger risks for biodiversity than fine woody debris extraction.

**Electronic supplementary material:**

The online version of this article (10.1007/s00442-019-04473-2) contains supplementary material, which is available to authorized users.

## Introduction

Intensive forestry modifies forest habitats, which has negative consequences for biodiversity. Emulation of natural disturbances has often been regarded as a key measure to make forestry more biodiversity-oriented (e.g., Hunter 1993; Nilsson and Ericson 1997; Tittler et al. 2012). Retention forestry, in which living and dead trees are retained at final harvesting, is an example of the application of that approach (Beese et al. 2003). Indeed, clearcuts with retention have frequently been found to be more species-rich than those without (Fedrowitz et al. 2014). However, since retention increases the structural diversity, such comparisons constitute only weak support for the hypothesis that emulation of natural disturbances being generally favourable for biodiversity; increased structural diversity may promote biodiversity independently of whether natural conditions are emulated or not. Thus, the hypothesis has rarely been tested based on adequate comparisons between habitats mainly formed by natural and anthropogenic disturbances.

The idea of emulating natural disturbances is based on the assumption that species communities are evolutionarily adapted to the conditions in their natural habitats (Bunnell 1995). Thus, the idea is related to the evolutionary species pool hypothesis, which suggests that most species are adapted to the naturally most abundant habitats (Taylor et al. 1990). This implies that more species are specialised to and able to utilize naturally more abundant habitats in comparison to rarer habitats. This is because more abundant habitats harbour communities of species with a larger total number of individuals, and a larger number of individuals mean that more species may occur in viable populations. The hypothesis has been confirmed at the global scale, correlating species richness with conditions over evolutionary scales (Pärtel 2002). However, it may also be valid for regional species pools resulting from immigration and regional species extinctions. The evolutionary species pool concept has been shown to explain species richness patterns in plant communities (e.g. Pärtel 2002; Schamp et al. 2002; Pither and Aarssen 2005; Hájek et al. 2007). The concept is important to consider in nature conservation, especially if the relationship at natural conditions would remain also after the habitat amounts have been changed due to anthropogenic impact, since then habitats which have decreased would have higher conservation value. However, to our knowledge, this has not been tested for any forest habitats.

Natural disturbances in forests, such as fires, storm fellings, and attacks by tree-killing species, typically result in dead wood, which is important for biodiversity. An estimated 20–25% of all forest species are dependent on dead wood (in Finland: Siitonen 2001). Intensive forestry may decrease the volume of logs and snags to only a tenth of natural volumes (Siitonen 2001), and therefore, the amount of dead wood has to be maintained or restored to preserve forest biodiversity. Species in dead wood have been shown to be associated with characteristics of individual dead-wood items, such as tree species, diameter, and position (standing/downed) (fungi: Berglund et al. 2011; Junninen and Komonen 2011; lichens: Humphrey et al. 2002; beetles: Ranius et al. 2015). For dead-wood habitats, the evolutionary species pool hypothesis suggests that dead-wood types utilized by most species are those formed by tree mortality factors that were most abundant under pre-human conditions, while dead-wood types to a higher extent created by forestry operations are used by fewer species, since such types have only recently become abundant. The extent to which species use these dead-wood types is important from a management and conservation perspective, because forest management for timber and pulp production results in a strong decrease of certain types of logs and snags (Siitonen 2001) while high volumes of stumps and fine woody debris (FWD) are formed. There is an increased interest to use stumps and FWD as a source of low-net-carbon energy, but there are concerns about the consequences of stump and FWD harvest on biodiversity (Hiron et al. 2017). Therefore, it is important to assess the conservation value of bioenergy wood in comparison to other dead wood.

We tested predictions based on the evolutionary species pool hypothesis on three species-rich groups of dead-wood-dependent organisms—fungi, lichens, and beetles. They are all intimately linked to the dead-wood items, which makes the evolutionary species pool hypothesis particularly relevant (Pither and Aarssen 2005). In a boreal forest landscape in central Sweden, we surveyed dead wood in all main types of forest stands. In this landscape, there is no sign of dispersal constraints in the distribution pattern of the great majority of all dead-wood-dependent beetle species and characteristics of dead-wood items have been found to explain beetle species’ occurrence patterns to a much higher extent than stand characteristics (Ranius et al. 2015). We relate species pool size to habitat extent in unmanaged forests, since they reflect historical conditions better than the managed forest stands that are currently dominant in the region. We have chosen a landscape dominated by managed forest, because it is especially if the species pool concept is valid under such conditions (i.e., that the species pool is a legacy reflecting older, more natural habitat amounts), it is important to consider in nature conservation. We divided the dead wood into four categories—(1) stumps and (2) FWD, which creation is favoured by forestry operations (Dahlberg et al. 2011), and (3) logs and (4) snags, which are more abundant in unmanaged forests as a result of natural tree mortality. This categorisation makes the outcome relevant when evaluating the effect of harvesting stumps and FWD. To be able to compare dead-wood types, we collected samples of organisms from specific dead-wood items. Our analyses consisted of the following four steps:

1. We compared the amount of different types of dead wood in managed and unmanaged forests. We assumed that the unmanaged forests mainly reflect the conditions resulting from natural disturbances, which the species pool is adapted to, and that the managed forests mainly reflect the conditions resulting from anthropogenic disturbances. We tested the following two predictions based on the evolutionary species pool concept.
2. In each species group, the number of species in each type of dead wood is positively related to the amount of dead wood of each type in unmanaged forests. This is because we assume that the number of species present is reflecting how many that are adapted to each habitat.
3. The habitat quality (measured as sum of relative population densities for all species, with the same weighting given to every species in the species pool) is higher in logs and snags than in FWD and stumps, due to that the former occur in higher abundances in unmanaged forests, because they are mainly formed by natural mortality and the latter by anthropogenic disturbances. Finally, we wanted to see whether the similarity in species richness among dead wood types which we observed in several cases was due to that the species communities were similar.
  Therefore:
4. We assessed the species similarity among dead-wood types for each species group.

## Methods

### Study landscape

All data were collected from a 24,449 ha area in the province of Hälsingland in central Sweden (62°N, 16°E). The studied landscape comprised a single block of land owned by one forest company, Holmen Skog AB. Of the total area, 20,294 ha were productive forest land (i.e., site productivity > 1 m^3^ha^−1^year^−1^) and the remaining parts mainly mires and lakes. The landscape is typical of the central boreal region (Ahti et al. 1968), with Scots pine (*Pinus sylvestris* L.) and Norway spruce [*Picea abies* (L.) Karst.] the dominant tree species. Birches (*Betula pendula* Roth and *B. pubescens* Ehrh.) are the most common deciduous tree species, but they generally only account for a minor part of the stand basal area. Since the 1950s, the forest has been managed more intensively and harvested by thinning and clearcutting. The landscape is now mainly composed of even-aged stands of ages representing the entire rotation period of about 100 years. Managed stands older than 60 years are remnants of the forest before clearcutting, though they have been affected by single tree harvest (historical high-grading) and thinning, while younger stands harbour one generation of trees growing after the last clearcutting. Within the study landscape, there are three large (average size: 250 ha), legally protected nature reserves (since the 90s), and about 375 smaller (average size: 5.1 ha, sometimes occurring contiguously to each other) voluntarily set-asides (according to the FSC certification standard) that together make up 13% of the forested area. Nature reserves and set-asides have been selected based on high conservation values, i.e., these forests are old and only little affected by humans. This means that they are not representative for stands recently affected by natural stand-replacing disturbances, but for stands affected by natural small-scaled disturbances. During the last 15 years, green tree retention has been applied at final harvest. Since the beginning of 2000, slash to be used for bioenergy has been harvested during clearcutting.

### Data collection

Field data were collected between 2001 and 2014 with the intention of being able to make comparisons. We included all types of productive forests, and stratified the sampling based on a classification of stands into four categories: “Clearcuts” stands for 3–14 years old, “Young forest” is 15–59 years old, “Old forest” is managed forest stands ≥ 60 years old, and “Unmanaged forest” is voluntary set-asides and nature reserves.

In 2001–2003, data on dead wood > 10 cm in diameter (except stumps) and dead-wood dependent beetles were collected (Ekbom et al. 2006; McGeoch et al. 2007). Additional data on dead-wood amounts were collected in 2009 and 2014 for stumps, and in 2013–2014 for FWD. Data on beetles were added, in 2004 for snags (Schroeder et al. 2006), in 2009 for stumps (Jonsell and Schroeder 2014) and in 2013 for FWD. Data on lichens and fungi in all types of wood were collected in 2013 (Hiron et al. 2017). The number of samples for each dead-wood category varied mainly in accordance with their abundance in the landscape (Online Appendix A), as well as the number of investigated stands (Online Appendix B).

### Dead-wood surveys

Dead wood was surveyed, applying a stratified random sampling in a subset of stands (Online Appendix B). The surveys for logs were performed with four transects of 100 m (two east–west and two north–south) in each stand (Ekbom et al. 2006). By extending the same transects to 10 m on each side, 0.2 ha sampling plots for snags were obtained. Stumps and FWD were surveyed in 100 m^2^ circular plots distributed in a regular quadratic network with 10–12 plots per stand (Jonsell and Schroeder 2014). For each dead-wood item surveyed, we collected data on tree species, dead-wood type, diameter (at 1.3 m for standing dead wood, top diameter for stumps, and basal diameter for downed dead wood), length or height and proportion of bark cover as follows: (1) Tree species: spruce, pine, or birches (i.e., the three most common tree species, other tree species, in total, constitute < 10% of all trees and were not considered). (2) Dead-wood type: stumps, FWD, logs, and snags. Standing dead-wood items with a diameter > 10 cm were defined as stumps if shorter than 50 cm and as snags if taller than 50 cm. Logs were defined as lying dead wood with a diameter > 10 cm. All dead wood with a diameter > 1 cm, but < 10 cm was FWD. FWD crushed by forestry machinery was not measured as this wood provides habitat of limited value for dead-wood-dependent organisms. All these four categories may include both naturally formed and anthropogenically created dead wood. Any dead-wood item can be divided into one of these categories; for instance, dead trees with branches left are categorised as snags or logs without considering the dead branches.

### Calculations of dead-wood quantities

For each stand, we estimated the amount of snags, stumps, and FWD per ha by dividing the amount found by the surveyed area. The amount of logs was estimated with a line intersect method (Marshall et al. 2000). We used three different measures of dead-wood amounts considered to be the most suitable for our three study organism groups; for fungi: dead-wood volume, for lichens: surface area of bark-free dead wood, and for beetles: area of dead wood covered by bark.

### Fungal survey

Fungal data were based on taxa detected and identified by DNA barcoding of wood samples. We studied only macrofungi (hereafter fungi), defined as all wood-inhabiting basidiomycete species known to form macroscopic (i.e., visible or > 1 mm) sporocarps, i.e., polypore, corticoid, and species in the order Agaricales. In each stand, dead-wood items were sampled at three positions 100 m apart at least 25 m from the stand edge. One drill dust sample was collected from the centre of the wood object (for stumps 10 cm below the cut, for snags at 1.3 m from the ground) using a 10-mm-diameter drill. Any bark and the surface layer of wood were removed with a knife prior to drilling to prevent sample contamination by surface-dwelling fungi and lichens. The sample covered almost the whole wood-piece diameter, taking care not to drill all the way through to the other side. The drill was carefully cleaned between sampling of different stumps by washing in water and ethanol. The drill dust samples were collected separately in plastic zip bags and kept frozen at − 20 °C until DNA extraction. All the field sampling was conducted in August and September 2013, and resulted in 596 samples. Fungi present in each dead-wood item were identified by Ion Torrent^TM^ sequencing of fungal DNA amplicons [see Hiron et al. (2017) for details]. Totally, we identified 1666 fungi-OTU, of which 104 were wood-inhabiting macrofungi and thus included in the study. Of all DNA, 28% belonged to these species.

### Lichen survey

Dead-wood dependent lichens (i.e., those solely occurring on wood without bark) were surveyed along the longest possible transect through each stand. Dead-wood objects with a surface area > 25 cm^2^ found within 5 m either side of the line walked were surveyed for lichens. If there were too few dead-wood items along the first transect, a second transect was inventoried. The surface of standing dead wood was surveyed up to a height of 2 m. Only lichen species that exclusively live on dead wood (without bark) according to Spribille et al. (2008) were included, with the exception of *Cladonia botrytes*, which was excluded as it has now been shown also to occur on other substrates. On each wood object, the area covered by each dead-wood-dependent lichen species was visually estimated in cm^2^. The surveyed area of dead wood without bark was calculated for each object based on collected data relating to length, circumference, and the percentage of bark.

### Beetle survey

Beetles were surveyed under the bark of dead wood by sifting bark in the field. We avoided dead wood from trees that recently had died (within 1 year), since they harbour a different beetle fauna. In this way, we collect beetles associated with specific dead wood items, but many species present in a dead-wood item are not collected by sieving at one single occasion (Wikars et al. 2005). In the laboratory, beetles were extracted from the resulting fine fraction using Tullgren funnels. For logs and snags, ten dead-wood items per stand were randomly selected and 0.5–1 m^2^ bark was sifted from each object (see McGeoch et al. 2007 for more details). For stumps, we took one 0.5 m^2^ sample of bark from each tree species (spruce, pine, and birch), with eight samples per tree species per stand (see Jonsell and Schroeder 2014 for details). If the required amount of bark was not available, all bark was sampled and the bark area noted. For FWD, we also took 0.5 m^2^ samples. All sampling of beetles was conducted during summer. Adults were identified to species and number of individuals per sample was counted by Stig Lundberg, Joel Hallqvist, Vitezslav Manak, and MJ. Only species dependent on dead wood according to Hansen (1964), Koch (1989)and Palm (1959) were included in our analyses.

### Statistical analyses

When evaluating effects of slash and stump harvesting, differences between the four dead-wood types are most important. To see whether the patterns obtained were consistent between tree species, we also examined each tree species separately.

First, we compared the volume of dead-wood types between managed forest and unmanaged forest. We calculated weighted mean volumes for each dead-wood type in managed and unmanaged forests, using sampled stands as replicates. The observations were weighted according to the number of sampled stands and the area of each stand type in the study landscape to obtain landscape-scale means. For each dead-wood type, we used a weighted *t* test to analyse whether the mean volumes differed between managed and unmanaged stands. All managed stands > 100 years were removed from the analysis, since these were older than the average rotation time for forest stands in Sweden and, therefore, not representative of stands affected by current management regimes. One stand classified as unmanaged but with a high volume of stumps from cutting was removed as well.

Second, we checked whether the number of species (comparing sample sizes standardized by rarefaction) was consistent with expectations based on the amount of the dead-wood type in unmanaged forest. We constructed sample-based rarefaction curves considering the dead-wood items as samples. For lichens and beetles, the *x*-axes were then re-scaled using the mean size of sampled dead-wood items, so that the number of species in each dead-wood type was plotted against the surveyed amount of dead wood. For fungi, the species number was plotted against the number of sampled dead-wood items, since, given the sampling method (one drill sample of the same size from each dead-wood item, regardless of its size), the samples were not deemed to represent the total volume of surveyed the dead-wood items. Species data were collected in both sun-exposed (disturbed) and shaded forest habitats, while dead-wood data in unmanaged conditions were collected only in shaded conditions. To control for the possible effect of this inconsistency, we also compared dead-wood data with species data from shaded forest habitat alone.

Third, we tested whether there were differences in habitat quality between the four dead-wood types, assuming that the density of individuals reflects habitat quality. To give all species the same weight, we used relative densities, which were calculated, so that for each species, the sum of densities on the four different dead-wood substrates was one. We standardized the sample sizes to be equal for each dead-wood type. For lichens and beetles, sample sizes were measured as the surveyed bark and wood area, and for fungi, as the number of surveyed dead-wood items. Using the dead-wood type with the smallest sample size as a base, we took 20 random subsamples of the same size for all other dead-wood types. The relative species densities were calculated as a mean of the 20 random samples. For lichens, density was calculated as the proportion of the surveyed surface area of wood covered by the species, and for beetles as the number of individuals observed per square meter of bark cover. For fungi, only presence–absence data at dead-wood item level were available, and the density was, therefore, estimated as the proportion of dead-wood items where the species was present. We only included species with five or more occurrences (occurrence = species observed on one dead-wood item), because, for rarer species, the uncertainties about their distribution among habitats become too large. Using the same data with standardized sample sizes, we tested statistically whether there were differences in the relative species density measure for each dead-wood type when each species were treated as a sample, first using a Kruskal–Wallis rank sum test and then Dunn’s test of multiple comparisons as a post hoc test. These are non-parametric rank tests, and thus appropriate to use, even though the response variables are relative densities.

Finally, we assessed whether the species-community composition differed among tree species and dead-wood types using Morisita–Horn similarity indices and PermANOVA tests. The similarities were calculated separately for each species group (fungi, lichens, and beetles) using data on species densities.

All statistical analyses were conducted using the statistical package R 3.5.0 (R Core Team 2018). The R-package “weights” (Pasek 2016) was used for the weighted *t* tests, package “iNEXT” (Hsieh et al. 2016) for constructing the rarefaction curves, package “Dunn. test” (Dinno 2017) for the Dunn tests, package RVAideMemoire (Hervé 2018) for the PermANOVAs, and the package “vegan” (Oksanen et al. 2017) for calculating dissimilarity indices.

## Results

**Fig. 1:**
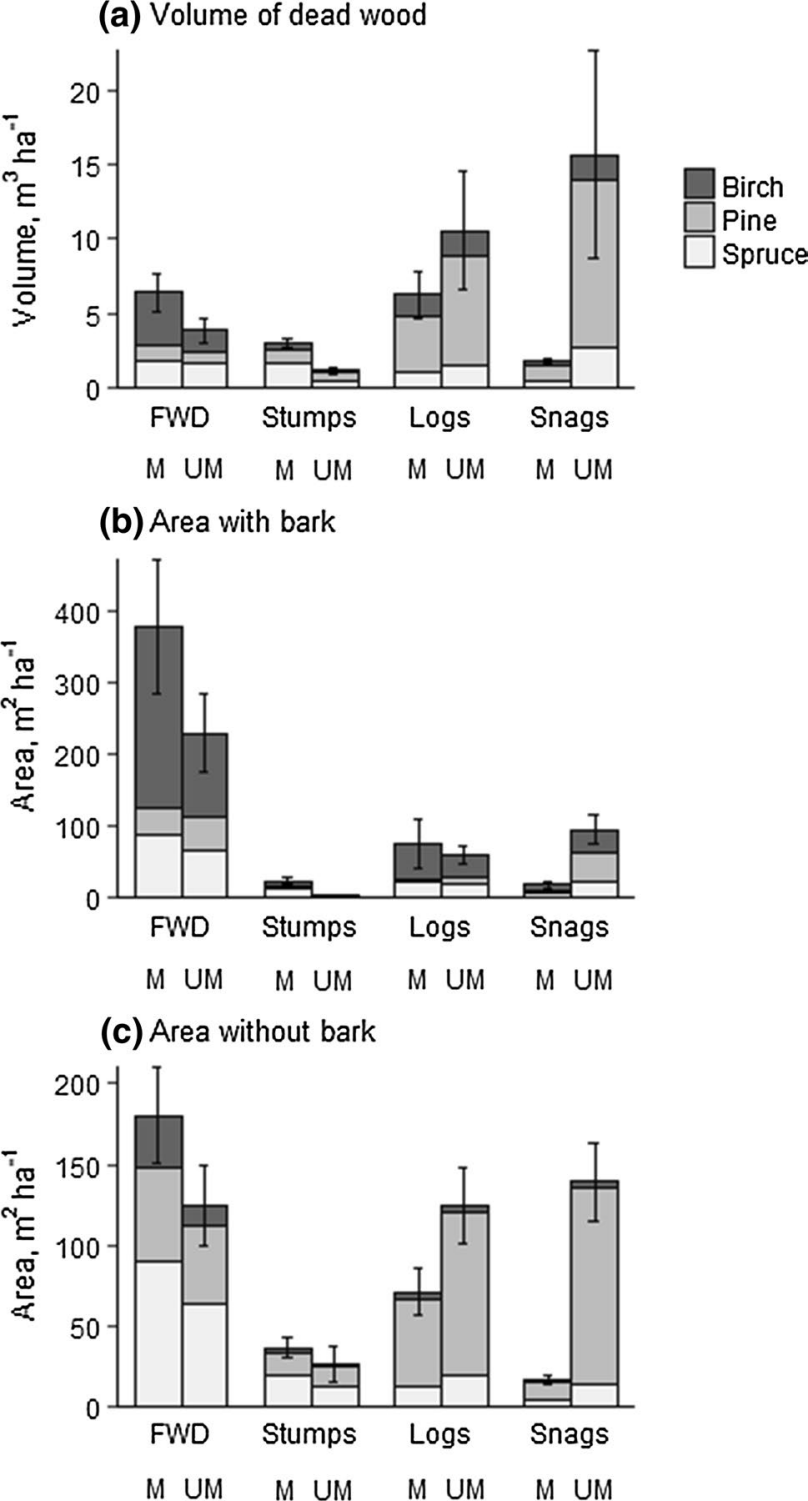
Quantity of dead wood of different types and tree species in managed and unmanaged stands. Means (± SE) weighted according to the proportion of different stand types at the landscape level. Statistical tests: see Table 1. The volume corresponds to the amount of habitat for fungi (**a**), surface area with bark for beetles (**b**), and surface area without bark for lichens (**c**)

The relative amounts of different dead-wood types depended on how the amount was measured (Fig. 1). In terms of volume, snags and logs were more abundant in unmanaged forest, while stumps and, to some extent, FWD (*P* = 0.06) were more abundant in managed forest (*P* < 0.05, weighted *t* tests; Table 1).

**Table 1:** Results of weighted *t* tests comparing the dead-wood volumes between managed and unmanaged forests (calculated separately for each dead-wood type and for each tree species as well as overall)

	FWD	Stumps	Logs	Snags
All dead wood	*t* = 1.98 *df* = 25.4 *P* = 0.06	*t* = 2.60 *df* = 24.3 ***P*** **= 0.02**	*t* = − 2.07 *df* = 50.0 **P = 0.04**	*t* = − 5.80 *df* = 35.9 **P < 0.001**
Pine	*t *= 0.61 *df* = 39.5 *P* = 0.55	*t* = 1.11 *df* = 13.1 *P* = 0.29	*t* = − 1.80 *df* = 47.7 *P* = 0.08	*t* = − 4.52 *df* = 35.4 ***P*** **< 0.001**
Spruce	*t* = 0.37 *df* = 15.6 *P* = 0.71	*t* = 2.01 *df* = 25.2 *P* = 0.06	*t* = − 1.20 *df* = 52.1 *P* = 0.24	*t* = − 1.96 *df* = 36.9 *P* = 0.06
Birch	*t* = 2.20 *df* = 41.3 *P* = 0.03	*t* = 2.51 *df* = 35.0 *P* = 0.02	*t* = − 0.33 *df* = 100.5 *P* = 0.74	*t* = − 3.27 *df* = 37.7 ***P*** **= 0.002**

Weighting was based on the area covered by different forest types in the study landscape. Statistically significant relationships are written in bold. Since the same hypotheses were tested for three tree species; for the latter 12 tests, significance thresholds was calculated based to Bonferroni sequential corrections

**Fig. 2:**
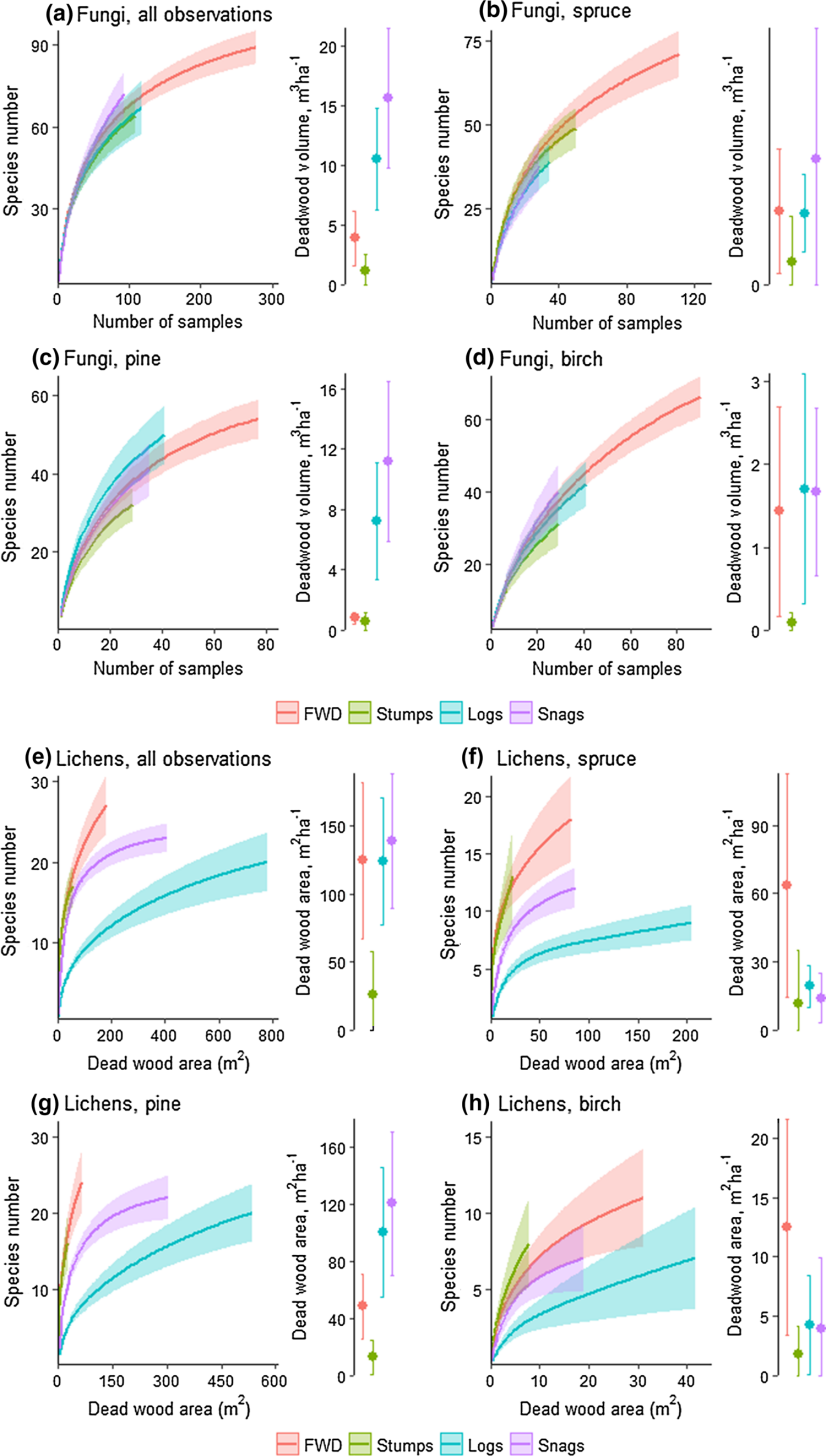

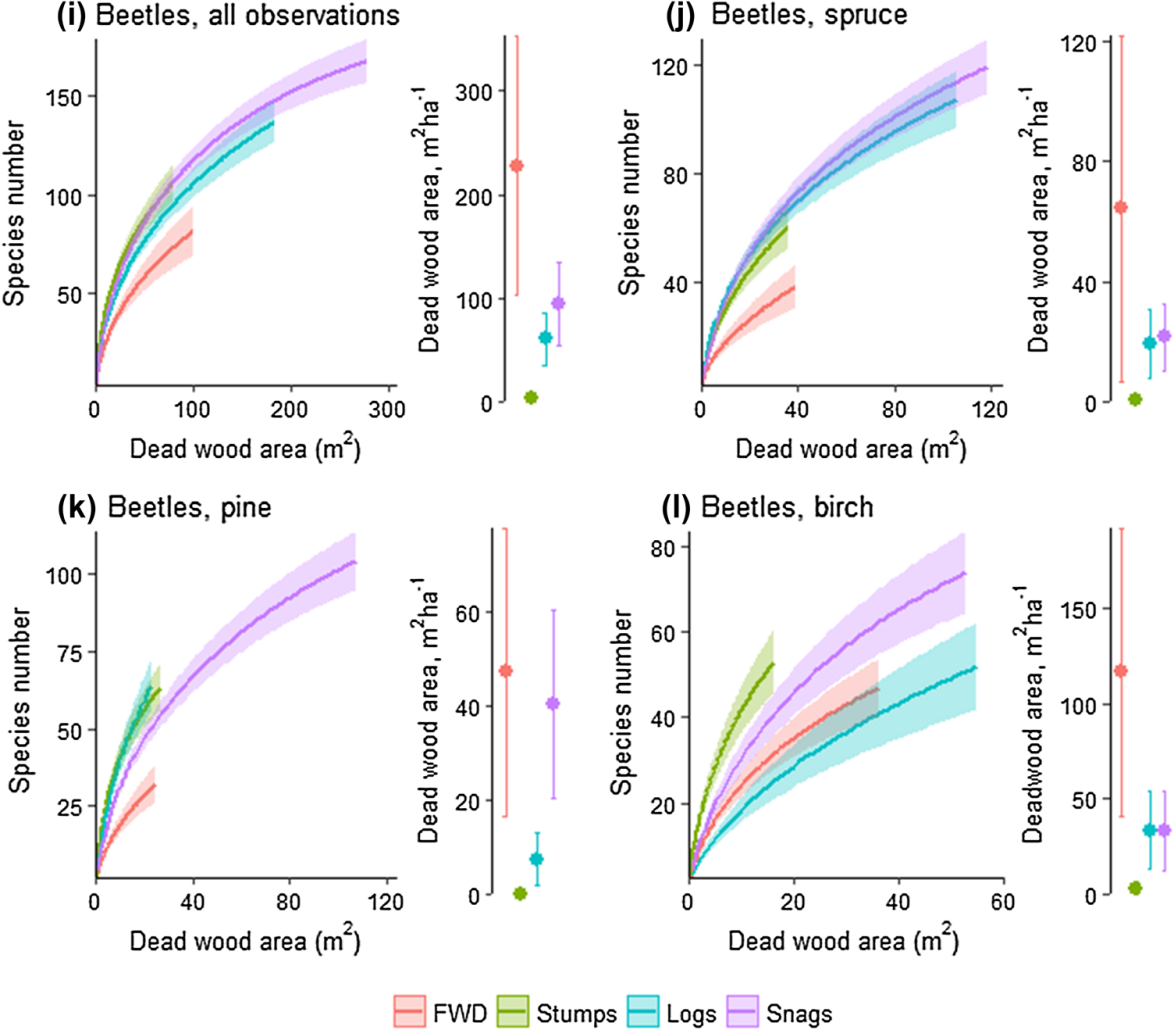
Test of the evolutionary species pool hypothesis by comparing species richness of various species groups in different dead-wood types with the amount of these dead-wood types in unmanaged forests. Mean values and a 95% confidence interval. Sample sizes were standardized by rarefaction based on the mean size of dead-wood items for each dead-wood type

We plotted rarefaction curves, reflecting the number of species, against amount of each dead-wood type present in unmanaged forests (Fig. 2). The outcome did not fit with our predictions based on the evolutionary species pool hypothesis for any of the three studied taxa groups. For fungi, the differences in species richness were small among dead-wood types. For lichens, high species richness in FWD and snags was consistent with the evolutionary species pool hypothesis, while the species richness in stumps was unexpectedly high given the low amounts of stumps. For beetles, the species richness was lowest for FWD and stumps were among the dead-wood types with the highest species richness, while the habitat amount was highest for FWD and lowest for stumps. To check the robustness of this outcome, we did the same test again after removing data from sun-exposed forest habitat (i.e., clearcuts). Then, the outcome still did not fit with predictions from the evolutionary species pool hypothesis; the only clear difference was that for beetles, stumps became less species rich (Online Appendix C).

**Fig. 3:**
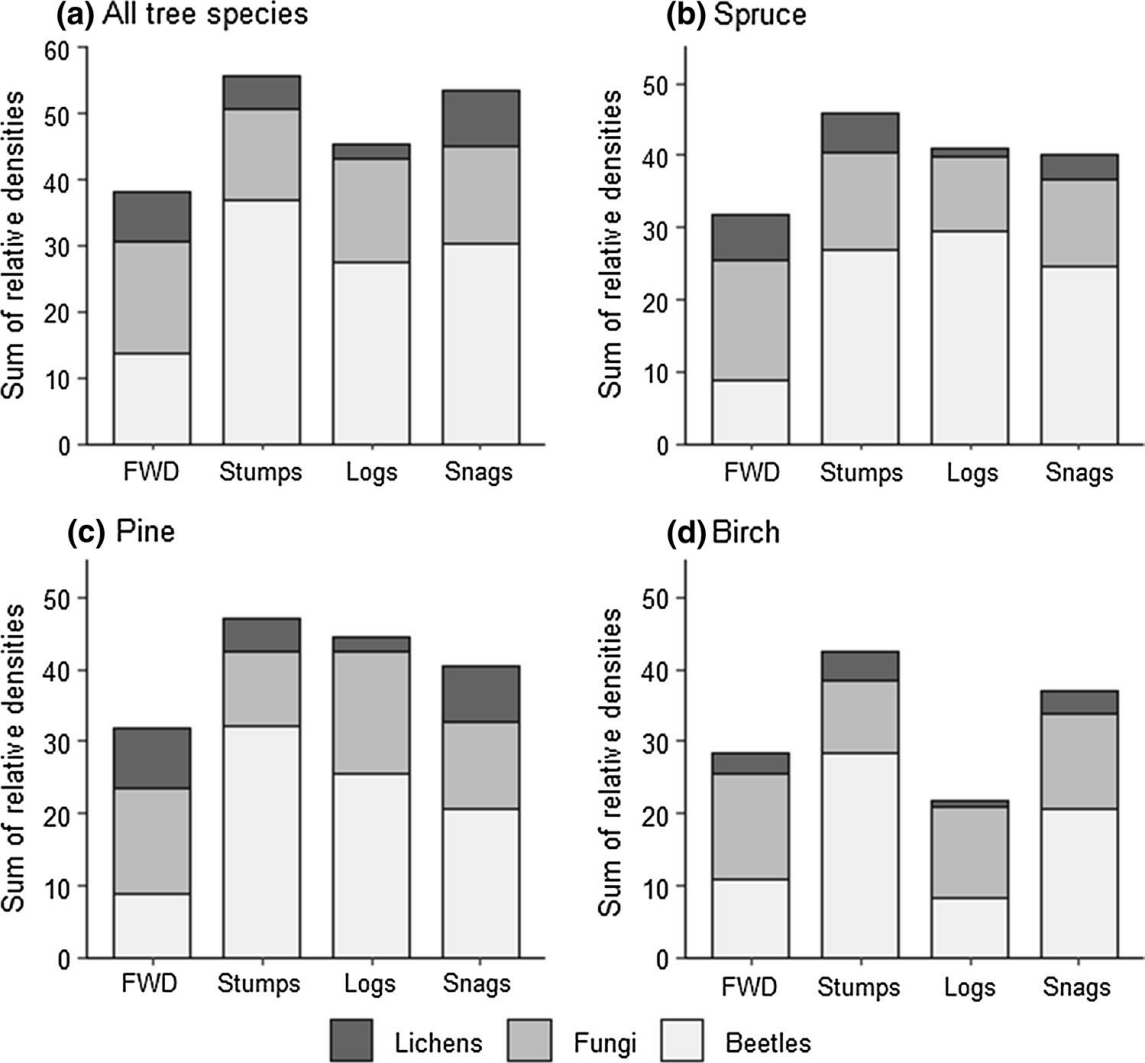
Habitat quality measured as the summed relative density of all species found in > 4 dead-wood items, with the same weight given to every species. **a** Total for all tree species and **b–d** calculated separately for pine, spruce, and birch. Outcomes from statistical tests are shown in Online Appendix D

For lichens and beetles, there were differences in total relative density of all species among the four dead-wood types (Fig. 3; Kruskal–Wallis rank sum test, *P* < 0.05). For fungi, significant differences were not observed (*P* = 0.27). Post hoc tests (Dunn’s test of multiple comparisons, *P* < 0.05) revealed that for beetles, FWD had lower habitat quality than the other three dead-wood types, and for lichens, snags and FWD had higher habitat quality than logs. When analysing each tree species separately, there were for fungi significant differences among dead-wood types (Online Appendix D).

The differences in species composition were significant or close to significant for pairs of dead-wood types for all taxa (Table 2). For fungi, the differences in species composition were smaller than for lichens and beetles. Raw data are presented in Online Appendix E and F.

**Table 2:** Morisita–Horn similarities in species composition between dead-wood types (all stand types combined and tree species combined)

	Snags	Logs	Stumps	Snags	Logs	Stumps
Fungi						
FWD	0.949	0.047	0.819	*P* = 0.03	*P* = 0.04	*P* = 0.02
Stumps	0.870	0.886		*P* = 0.02	*P* = 0.02	
Logs	0.930			*P* = 0.09		
Lichens						
FWD	0.071	0.350	0.934	*P* = 0.01	*P* = 0.01	*P* = 0.02
Stumps	0.211	0.472		*P* = 0.01	P = 0.01	
Logs	0.929			*P* = 0.01		
Beetles						
FWD	0.220	0.087	0.318	*P* = 0.01	*P* = 0.01	*P* = 0.01
Stumps	0.282	0.042		*P* = 0.01	*P* = 0.01	
Logs	0.539			*P* = 0.01		

*P* values from PermANOVA testing the probability that the observed pattern would occur if the species composition was the same. For comparisons among all substrate types, *P* = 0.01 for all three groups

## Discussion

### Dead-wood amounts in managed and unmanaged forests

The difference in amounts of dead wood between managed and unmanaged forests was significant, but small compared to data reported in other studies, which have estimated volumes of logs and snags in managed forests corresponding to only 2–10% of the volumes in old growth forests (Siitonen 2001). The smaller difference observed is probably due to two factors. First, forest management has been intensified lately (during the last 60 years) in our study landscape, and in managed forests, living and dead trees are, therefore, still remaining as legacies from more natural conditions. Consequently, in the managed forests in our study area, the volume of logs and snags is about twice as high as the average for managed forests in Sweden (Ekbom et al. 2006). Second, the unmanaged forests were set aside relatively recently, and trees may have previously been harvested by selective cutting in them. This explains why the volume of snags and logs is just a third of the average measured in reserves in northern Sweden (Linder et al. 1997).

Both FWD and stumps were more abundant in managed than in unmanaged forests. These types of dead wood have rarely been surveyed outside clearcuts (however, see Hagermann et al. 2009), but our results show that they are important to consider especially in managed forests, since they constitute a large part of the total dead-wood volume (Fig. 1), and are also utilized by many dead-wood-dependent organisms (Fig. 3). The abundance of these dead-wood types is strongly influenced by harvesting of slash and stumps for bioenergy. In our study landscape, during recent years, slash has been harvested from up to 50% of the clearcuts, while stumps have not been harvested (Hiron et al. 2017).

In summary, forest management for timber and pulp production decreases the amount of naturally formed dead wood. However, since it increases the amount of stumps and FWD, the total decrease is not as large as it appears when only data for logs and snags (including any standing or downed dead trees) are reported, which often is the case.

### Evolutionary species pool hypothesis

Overall, we found no support for the evolutionary species pool hypothesis for any of the studied species groups (Fig. 2). The inconsistency for beetles can only be partly explained by the fact that the area covered with bark does not perfectly reflect the amount of habitat. Indeed, the area tends to overestimate the amount of FWD, since the phloem thickness increases with the size of dead-wood items (Hölttä et al. 2013). However, the difference is rather small; the ratio between phloem volume and area covered by bark and is only 20% higher for a 30-cm-diameter dead-wood item in comparison to a 5-cm-diameter item (calculated from Hölttä et al. 2013).

The fact that beetle and lichen species richness was not consistent with the evolutionary species pool hypothesis can be explained by biophysical characteristics of the dead-wood types, i.e., the physical and biotic conditions such as moisture, temperature, food supply, and longevity. The low beetle numbers in FWD could be because there are not enough resources for larval development in the FWD items, the microclimatic conditions are highly variable, and the habitat is short-lived. In comparison to other dead-wood types, stumps have thicker bark and their connection to the ground may make them less prone to drying out, which may favour both lichens and beetles. For that reason, species adapted, through evolution, to microhabitats in any part of a tree may occur in high densities in stumps.

We found no clear differences among any dead-wood types with respect to fungal species richness (Fig. 2). Regarding community composition, the differences were also weaker for fungi in comparing to lichens and beetles, but in most cases still statistically significant (Table 2). This is probably not because these species are generally little affected by any dead-wood characteristics, but at least partly because species are influenced by other dead-wood characteristics than those recognized in our categorisation. This means that even though the evolutionary species pool concept is valid in theory, it may still be difficult to apply due to a lack of knowledge about how species communities divide their use of habitats.

### Habitat quality of dead-wood types: consequences for stump and FWD harvest

Snags, stumps, and logs were of higher habitat quality (measured as relative density of species) for dead-wood dependent organisms than FWD (Fig. 3). Given the high habitat quality of stumps, there was overall no clear difference in habitat quality between mainly natural and mainly anthropogenic dead-wood types. The great majority of our surveyed stumps were man-made, which was also the case in general for stumps in the study landscape. At a smaller (dead-wood item) scale, Jonsell et al. (2004) and Pasanen et al. (2018) also found only minor differences in species richness between naturally formed and cut dead wood. However, they revealed differences in beetle species composition between dead-wood categories (Jonsell et al. 2004), and reported that the variation in fungal species composition among dead-wood items was higher in naturally formed dead wood (Pasanen et al. 2018), which is not consistent with the outcomes from our analyses (Fig. 2). Our study shows that the conservation value is not generally lower for mainly man-made dead-wood types. Note, however, that both our and other studies focus on one certain landscape each and their specific species pools. It would be interesting to assess many landscapes to test whether landscape composition and landscape history may affect the outcome.

Many species inhabiting large-diameter logs and snags are on national red lists, since these dead-wood types have decreased due to forestry for timber and pulp production (Tikkanen et al. 2006; Seibold et al. 2015). Recently, the extraction of FWD and stumps has increased, since they are sources of bioenergy (Lundborg 1998). There are concerns about how this affects biodiversity, since harvesting of FWD and stumps means that dead wood is removed (Hiron et al. 2017). We found that FWD is of lower habitat quality than other types of dead wood (Fig. 3). This is in line with earlier studies showing that large-diameter dead-wood items are used by more species than small-diameter items (Junninen and Komonen 2011; Ranius et al. 2015), even though FWD, especially of deciduous tree species, has been found to harbour beetle species considered to be of conservation concern (Jonsell et al. 2007). Also, according to expert opinions (Dahlberg et al. 2011) and field studies on lichens (Svensson et al. 2016), FWD represents a lower quality habitat than other dead-wood types. Stumps, on the other hand, were found to be of at least equal habitat quality to other dead-wood types (Fig. 3). Stumps have also previously been found to host species-rich communities of fungi (Kubart et al. 2016), lichens (Svensson et al. 2016), and beetles (Andersson et al. 2015; Brin et al. 2013; Jonsell and Hansson 2011). Thus, for biodiversity, stump extraction is associated with larger risks than FWD extraction. At a landscape scale, FWD in stands up to 20-year-old constituted 4–17% (depending on how dead wood is measured) and stumps in these stands represented 2–6% of all dead wood (Hiron et al. 2017). Consequently, even though many species occur in FWD and stumps, for most species, only a small part of the landscape-level habitat is affected by slash and stump extraction. However, 17% of all species had > 50% of their landscape-level populations in bioenergy wood, for instance, because they are associated with sun-exposed conditions (Hiron et al. 2017). For some of them, an extensive FWD and stump harvest may mean risks for extinction at a landscape level.

### Conclusions: emulating natural disturbances

Large-scale changes in forestry, aimed at emulating natural disturbances, have been conducted without testing whether they are effective (Larsson and Danell 2001), and the tests showing that these changes had positive effects on biodiversity came later (e.g., Fedrowitz et al. 2014). The idea that natural disturbances should be emulated in management and conservation efforts is connected with the evolutionary species pool concept. We have identified two reasons why the concept may not hold in many cases. First, any estimate of the historical amount of habitat relies on some habitat categorisation, and what seems to be a relevant categorisation for humans may not necessarily be relevant for inhabiting species. Second, biophysical conditions may modify or override relationships expected according to the evolutionary species pool concept. Thus, even if emulation of natural disturbances seems to be an appropriate approach in theory, empirical studies of entire species communities (such as, e.g., Lindhe and Lindelöw 2004; McMullin et al. 2013) are necessary to understand how natural habitats and disturbances should be emulated.

## Electronic supplementary material

Below is the link to the electronic supplementary material.

Supplementary material 1 (DOCX 134 kb)

Supplementary material 2 (XLSX 2023 kb)

Supplementary material 3 (XLSX 135 kb)
