# Supplementary material for: The evolutionary species pool concept does not explain occurrence patterns of dead-wood-dependent organisms: implications for logging residue extraction
Source: Oecologia. 2019 Jul 27;191(1):241–52. doi: 10.1007/s00442-019-04473-2 (PMC6732131; doi:10.1007/s00442-019-04473-2)
Supplement: Supplementary file 1 — Supplementary material 1 (DOCX 134 kb) [file 442_2019_4473_MOESM1_ESM.docx]

Appendix A. Number of samples for each substrate/tree species category used to calculate dead wood amounts and population densities.

|  | Stumps | | | FWD | | | Snags | | | Logs | | |
| --- | --- | --- | --- | --- | --- | --- | --- | --- | --- | --- | --- | --- |
|  | Spruce | Pine | Birch | Spruce | Pine | Birch | Spruce | Pine | Birch | Spruce | Pine | Birch |
| Dead wood |  |  |  |  |  |  |  |  |  |  |  |  |
| Clearcuts | 157 | 84 | 44 | 207 | 90 | 110 | 61 | 82 | 25 | 71 | 61 | 47 |
| Young forest | 22 | 23 | 28 | 93 | 56 | 86 | 2 | 31 | 4 | 5 | 23 | 11 |
| Old forest | 20 | 7 | 10 | 95 | 45 | 106 | 42 | 75 | 59 | 45 | 41 | 31 |
| Unman. forest | 15 | 12 | 5 | 79 | 82 | 82 | 57 | 104 | 78 | 36 | 70 | 30 |
| Fungi |  |  |  |  |  |  |  |  |  |  |  |  |
| Clearcuts | 35 | 15 | 15 | 36 | 27 | 35 | 13 | 12 | 9 | 14 | 14 | 14 |
| Young forest | 9 | 9 | 9 | 24 | 17 | 18 | 4 | 4 | 2 | 8 | 8 | 9 |
| Old forest | 6 | 5 | 4 | 25 | 16 | 20 | 7 | 7 | 7 | 10 | 7 | 9 |
| Unman. forest | 0 | 0 | 1 | 26 | 17 | 17 | 5 | 12 | 11 | 3 | 12 | 9 |
|  |  |  |  |  |  |  |  |  |  |  |  |  |
| Lichens |  |  |  |  |  |  |  |  |  |  |  |  |
| Clearcuts | 46 | 46 | 39 | 179 | 181 | 173 | 49 | 47 | 36 | 46 | 48 | 46 |
| Young forest | 21 | 20 | 18 | 92 | 84 | 87 | 12 | 13 | 10 | 19 | 17 | 21 |
| Old forest | 10 | 13 | 16 | 76 | 65 | 85 | 14 | 13 | 23 | 22 | 13 | 25 |
| Unman. forest | 20 | 18 | 7 | 82 | 94 | 92 | 21 | 25 | 26 | 23 | 25 | 25 |
|  |  |  |  |  |  |  |  |  |  |  |  |  |
| Beetles |  |  |  |  |  |  |  |  |  |  |  |  |
| Clearcuts | 74 | 73 | 39 | 36 | 9 | 28 | 82 | 11 | 9 | 26 | 4 | 14 |
| Young forest | 7 | 5 | 10 | 2 | 0 | 0 | 0 | 0 | 0 | 0 | 0 | 0 |
| Old forest | 39 | 40 | 32 | 5 | 7 | 12 | 60 | 48 | 39 | 50 | 10 | 12 |
| Unman. forest | 0 | 0 | 0 | 10 | 12 | 7 | 32 | 68 | 20 | 48 | 9 | 29 |
|  |  |  |  |  |  |  |  |  |  |  |  |  |

Appendix B. Number of stands with surveyed dead wood items.

|  |  | |  |  | | | Dead wood type | | |  | |  |
| --- | --- | --- | --- | --- | --- | --- | --- | --- | --- | --- | --- | --- |
| Dead Wood | | **Stand Type** | | |  | **Stump** | | **FWD** | **Snag** | | **Log** | |
|  | | Clearcuts | | |  | 18 | | 20 | 55 | | 55 | |
|  | | Young forest | | |  | 8 | | 10 | 20 | | 20 | |
|  | | Old forest | | |  | 6 | | 8 | 21 | | 21 | |
|  | | Unmanaged forest | | |  | 5 | | 9 | 36 | | 36 | |
|  | |  | | |  |  | |  |  | |  | |
| Fungi | |  | | |  |  | |  |  | |  | |
|  | | Clearcuts | | |  | 6 | | 6 | 6 | | 6 | |
|  | | Young forest | | |  | 3 | | 3 | 2 | | 3 | |
|  | | Old forest | | |  | 2 | | 3 | 3 | | 3 | |
|  | | Unmanaged forest | | |  | 1 | | 3 | 3 | | 3 | |
| Lichens | |  | | |  |  | |  |  | |  | |
|  | | Clearcuts | | |  | 11 | | 11 | 11 | | 11 | |
|  | | Young forest | | |  | 5 | | 5 | 5 | | 5 | |
|  | | Old forest | | |  | 5 | | 5 | 5 | | 5 | |
|  | | Unmanaged forest | | |  | 6 | | 6 | 6 | | 6 | |
| Beetles | |  | | |  |  | |  |  | |  | |
|  | | Clearcuts | | |  | 10 | | 11 | 28 | | 16 | |
|  | | Young forest | | |  | 5 | | 8 | 25 | | 23 | |
|  | | Old forest | | |  | 5 | | 4 | 25 | | 21 | |
|  | | Unmanaged forest | | |  | 0 | | 5 | 22 | | 19 | |

Appendix C. Test of the Evolutionary Species Pool Hypothesis by comparing species richness of various species groups in different dead wood types (including all forest habitats except clearcuts) with the amount of these dead wood types in unmanaged forests. Mean values and a 95% confidence interval. Sample sizes were standardized by rarefaction based on the mean size of dead wood items for each dead wood type. This test is as in Fig. 2, but aimed at controlling the robustness by excluding sun exposed (disturbed) forest habitat, i.e. clearcuts.


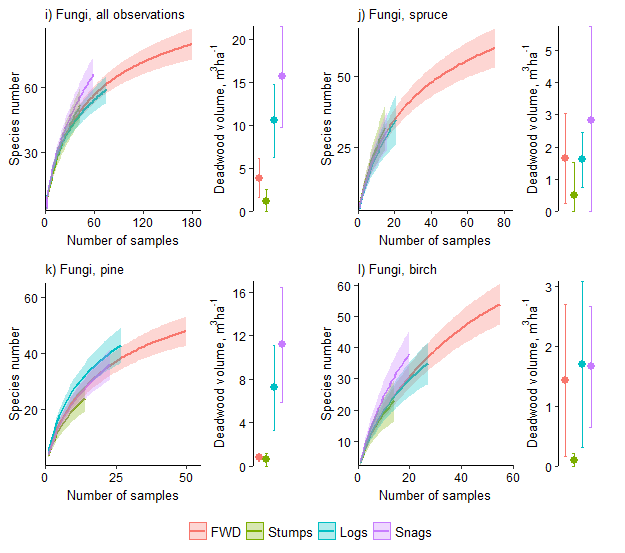


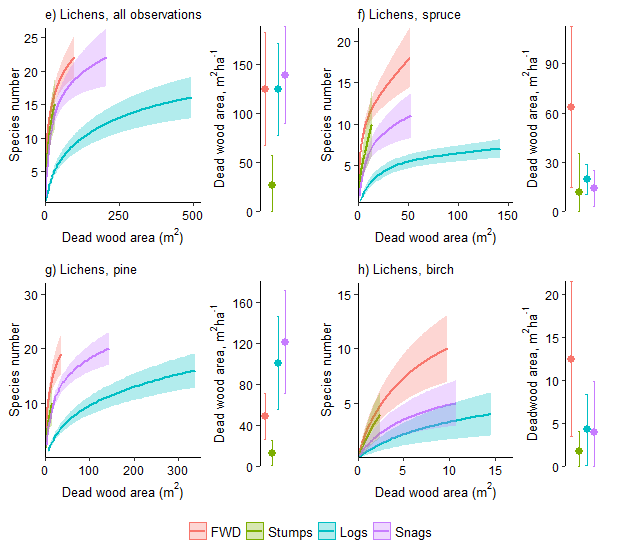


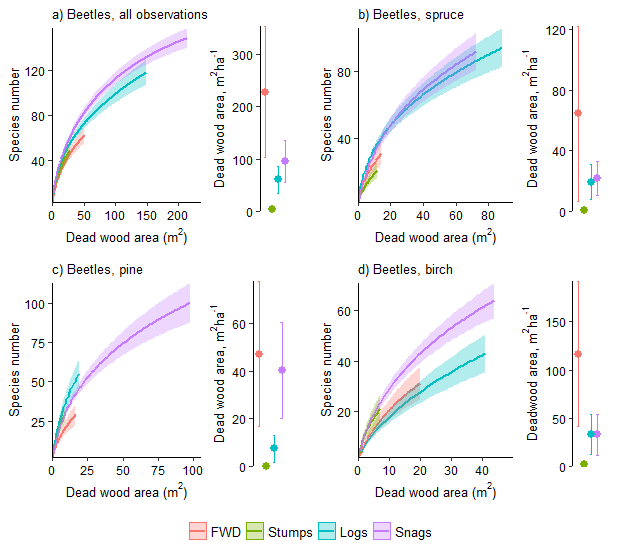


Appendix D. Comparison of species densities among substrate types (Kruskal-Wallis rank sum test and Dunn test for pairwise comparisons). Statistically significant relationships are written in bold. Since the same hypotheses were tested for three tree species; for the Kruskall-Wallis rank sum test, significance thresholds was calculated based to Bonferroni sequential corrections.

|  | **Whole data** | **Spruce** | **Pine** | **Birch** |
| --- | --- | --- | --- | --- |
| **Fungi** |  |  |  |  |
| Kruskal-Wallis rank sum test | χ² = 3.93  P = 0.27 | χ² = 9.63  **P = 0.02** | χ² = 12.17  **P = 0.01** | χ² = 6.84  P = 0.08 |
| Pairwise comparisons |  |  |  |  |
| FWD vs logs | - | **0.003** | 0.20 | - |
| FWD vs snags | - | **0.005** | 0.09 | - |
| FWD vs stumps | - | **0.04** | **0.008** | - |
| Logs vs snags | - | 0.42 | **0.02** | - |
| Logs vs stumps | - | 0.16 | **0.006** | - |
| Snags vs stumps | - | 0.22 | 0.15 | - |
| **Lichens** |  |  |  |  |
| Kruskal-Wallis rank sum test | χ² = 10.72  **P = 0.01** | χ² = 8.66  P = 0.03 | χ² = 9.49  P = 0.02 | χ² = 4.94  P = 0.18 |
| Pairwise comparisons |  |  |  |  |
| FWD vs logs | **0.004** | - | - | - |
| FWD vs snags | 0.45 | - | - | - |
| FWD vs stumps | 0.07 | - | - | - |
| Logs vs snags | **0.002** | - | - | - |
| Logs vs stumps | 0.11 | - | - | - |
| Snags vs stumps | 0.06 | - | - | - |
| **Beetles** |  |  |  |  |
| Kruskal-Wallis rank sum test | χ² = 35.06  **P < 0.001** | χ² = 54.33  **P < 0.001** | χ² = 35.46  **P < 0.001** | χ² = 17.82  **P < 0.001** |
| Pairwise comparisons |  |  |  |  |
| FWD vs logs | **< 0.001** | **< 0.001** | 0.20 | 0.46 |
| FWD vs snags | **< 0.001** | **< 0.001** | 0.09 | **0.001** |
| FWD vs stumps | **< 0.001** | **< 0.001** | **0.01** | **0.002** |
| Logs vs snags | 0.12 | 0.44 | **0.02** | **< 0.001** |
| Logs vs stumps | 0.42 | **0.01** | **< 0.001** | **0.002** |
| Snags vs stumps | 0.16 | **0.01** | 0.15 | 0.41 |
|  |  |  |  |  |
